# Long-term large-scale decline in relative abundances of butterfly and burnet moth species across south-western Germany

**DOI:** 10.1038/s41598-019-51424-1

**Published:** 2019-10-17

**Authors:** Jan Christian Habel, Robert Trusch, Thomas Schmitt, Michael Ochse, Werner Ulrich

**Affiliations:** 1 Evolutionary Zoology Group, Department of Biosciences, University of Salzburg, A-5020 Salzburg, Austria; 2 Terrestrial Ecology Research Group, Department of Ecology and Ecosystem Management, School of Life Science Weihenstephan, Technische Universität München, D-85354 Freising, Germany; 3 Department of Entomology, State Museum of Natural History Karlsruhe, D-76131 Karlsruhe, Germany; 4 Senckenberg German Entomological Institute, D-15374 Müncheberg, Germany; 5 Entomology, Zoology, Biological Institute, Faculty of Natural Sciences I, Martin Luther University Halle-Wittenberg, D-06099 Halle an der Saale, Germany; 6 Waldstraße 51, D-67273 Weisenheim am Berg, Germany; 7 Department of Ecology and Biogeography, Nicolaus Copernicus University Toruń, 87-100 Toruń, Poland

**Keywords:** Biodiversity, Environmental impact

## Abstract

Current studies have shown a severe general decline in insect species diversity, their abundance, and a biomass reduction of flying insects. Most of previous studies have been performed at single sites, or were spatially restricted at the landscape level. In this study, we analyse trends of species richness and shifts in species composition of butterflies and burnet moth species across the federal state of Baden-Württemberg in south-western Germany, covering an area of 35,750 km^2^. The data set consists of 233,474 records and covers a period from 1750 until today. We grouped species according to their species´ specific functional traits and analyse how species with different habitat requirements and behaviour respond to land-use changes over time. Our data document a significant loss of relative abundance for most species, especially since the 1950s until today. Species demanding specific habitat requirements are more seriously suffering under this trend than generalists. This in particular affects taxa adapted to extensively used xerothermic grasslands, bogs or other habitats maintained by traditional low-productivity agricultural practices of the past. Our data indicate large-scale decline in relative abundance of many butterfly and burnet moth species, which happened in particular during the past few decades.

## Introduction

Insect diversity declined over major parts of Europe during the past years^1^. Thomas *et al*.^2^ reported a reduction in species richness of butterflies across the UK, and other studies have indicated temporal shifts in species composition, accompanied by a loss of species richness and a decrease of species evenness^3^. Similar negative trends have also been observed for wild bees^4^ and carabid beetles^5^, observable at a larger scale, outside and inside of nature reserves^3,6^. Apart from decreasing species richness and shifts in species composition, recent studies also documented a severe reduction in arthropod abundances during the past decades^7^. In a widely commented study, Hallmann *et al*.^8^ identified a 75% loss of biomass of flying insects in western Germany during the past 30 years. Such losses of biomass, species richness and changes in species composition may have negative cascading effects to higher trophic levels. Recent studies confirmed that birds and bats^9^, which directly depend on the availability of insects as food source, also decreased significantly during the past decades.

Potential reasons driving this insect decline are manifold. Agricultural intensification is assumed to be the main reason, causing dramatic losses of habitats^10,11^, which leads to increasing isolation among the remaining habitats, with negative effects on the persistence of species at each single site^12^. Large monocultures pose hostile environments and create barriers for many species, further aggravating exchanges of individuals and thus population persistence^13^. In addition, decreasing general habitat quality, e.g. due to atmospheric nitrogen loads^14^ and drifting pesticides^15^, might have further negative effects on individuals, species and entire communities, particularly on species with specific ecological demands and hence limited potential to adapt^12^.

To survey the current status of biodiversity, analyses of population trends and potential shifts in community assemblies are needed. Long-term data are crucial to distinguish short term population fluctuations (with negligible long-term effects) from long-term population trends^16^. This is in particular of high relevance for groups of arthropods, which are known to fluctuate severely among generations^17^. There already exist various studies documenting loss of species richness, abundance, biomass, and shifts in species composition^1–8^. Apart from very few studies in which German-wide data sets were used^18,19^, most research projects were conducted on single taxa, do represent the situation of a geographically restricted area (i.e. one or some few sites) and mostly cover relatively short periods of time (few years to few decades, represented by two or few time steps)^5,6,8^. This makes most studies assailable, and the explanatory power remains limited and applies to a specific area and/or species.

In this study, we analyse long-term and large-scale data on butterflies and burnet moth species that have been collected across the federal state of Baden-Württemberg in south-western Germany, covering a total area of 35,750 km^2^. The data consist of 233,474 independent observations (i.e. single records, with a record representing one observation of a species in one 35 km^2^ grid cell during one year) and cover a period of 268 years. We classified each species according to its ecological requirements and behaviour (i.e. habitat demands, larval food plants used, and dispersal behaviour) to test for potential responses on land-use intensification. Based on our data, we address the following questions:

(i) Do butterfly and burnet moth species richness and relative abundance decline over time?
(ii) Which functional traits explain trends in species richness and abundances?
(iii) Do periods that are characterised by major species community shifts and changes of species richness and abundances coincide with periods which are known for major shifts in land-use intensification?

## Results

### Species richness

Of the 155 butterfly and burnet moth species known for Baden-Württemberg in total, 123 species were recorded during the 18^th^ century, 140 species during the 19^th^ century, 153 species during the first and the second half of the 20^th^ century, and 152 species during the 21^st^ century. All species recorded before 1800 were also present during the 19^th^, 20^th^, and 21^st^ century. There were no species exclusively found in one single 50 year sampling window. Neither numbers nor the proportion of specialists and generalists shifted over time (Table 1).

**Table 1:** Species richness, species gains and species losses, proportion of habitat generalists and habitat specialists for six time windows.

Factor	≤1800	1801–1850	1851–1900	1901–1950	1951–2000	>2001
Total species richness	123	129	129	153	153	152
Species only present in	0	0	0	0	0	0
Species only present before	—	0	0	0	2	1
Number of newly recorded species	—	16	5	9	2	0
Number of species not further recorded	—	0	0	0	0	3
Habitat generalists	56	54	57	60	62	62
Habitat specialists	67	75	72	93	91	90
Total number of records	739	683	1,036	12,158	79,467	138,529

Given is also the total number of records. Note that data are not corrected for the differential number of records.

**Figure 1:**
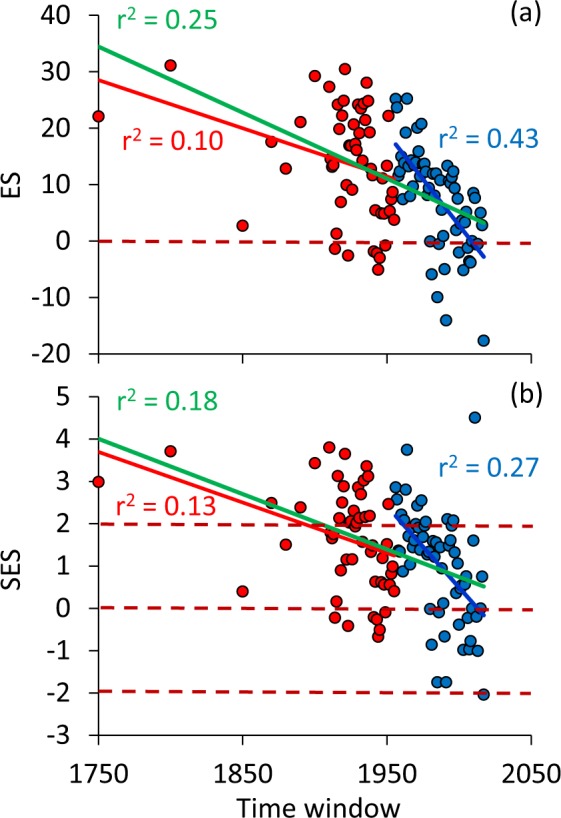
Analyses of (**a**) effect sizes (ES = S_obs_ − S_exp_) and (**b**) standardized effect sizes (SES) of species richness in each study window returned a breakpoint in 1956 (red data and regression line before, blue data and regression line since 1956). The green regression lines refer to all study windows. Explained variances (r^2^) refer to ordinary linear least squares regressions. All regressions are significant at P < 0.01. Broken lines define the zero effects and the upper and lower two-sided 95% confidence limits of SES.

**Figure 2:**
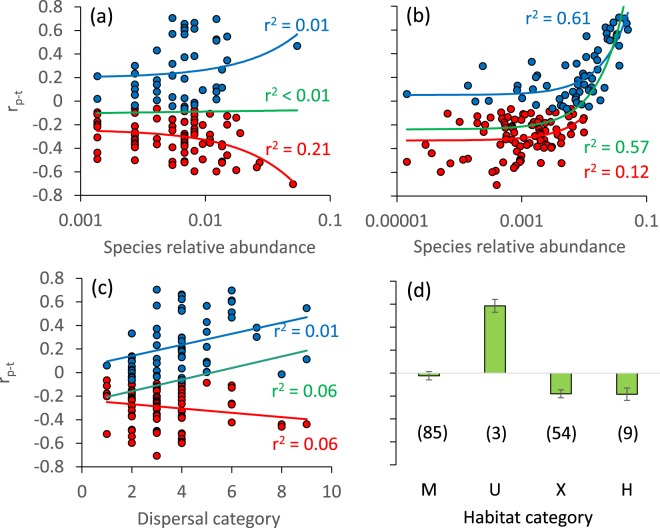
Correlations r_p-t_ of relative species abundance and the time window studied of each butterfly species in dependence on the relative abundance in the 18^th^ century (**a**), in the 21^st^ century (**b**), species’ dispersal (**c**, 1: lowest, 9: highest dispersal), and the habitat preferences (**d**, M: mesophilous, U: ubiquistic, X: xerophilous, H: hygrophilous). Blue data points and regression lines: generalist species; red data points and regression lines: specialists. The green bars and regression lines refer to all species combined. Errors in **d**) refer to standard errors, numbers in brackets to the number of species. Explained variances (r^2^) refer to ordinary linear least squares regressions.

The random sample approach of species richness revealed significant temporal trends of effect sizes ES (Fig. 1a) and standardized effect sizes SES (Fig. 1b). Breakpoint analyses pointed to only moderate changes in richness ES and SES up to 1955 (Fig. 1a,b). These trends were also detectable when excluding the data from the 18^th^ century (r^2^ = 0.06, P = 0.08). On the contrary, ES and SES strongly decreased after 1956 (Fig. 1a,b). In 92 of the 115 study windows, ES and SES were positive so that more species were observed than expected from a random sample (Fig. 1a,b). 36 standardized effects sizes were significantly positive at the 5% error level (Fig. 2b). Only seven of the 55 windows before 1956 were negative, while later 12 of the 60 effect sizes were so. Importantly, the tendency for positive effect sizes remained after controlling for the temporal differences in sample size (GLM: time window: partial η^2^ = 0.16, P = 0.001).

### Relative abundance

In a second approach, we tested for temporal trends in the relative abundance of each species in relation to habitat requirements and the Ellenberg indicator values of the main larval food plants used by the respective butterfly and burnet moth species (Table 2, ES1A,B, Fig. 2). Except for family membership and the degree of specialization (Table 2), the temporal trends in relative abundance were not clearly linked with any species trait. Thus, Lycaenidae (average r_p-t_ = −0.14 ± 0.03; mean ± standard error), Papilionidae (r_p-t_ = −0.34 ± 0.23), Zygaenidae, (r_p-t_ = −0.08 ± 0.03), and Hesperiidae (r_p-t_ = −0.12 ± 0.06) were the families with negative trends, while Pieridae increased in average relative abundance (r_p-t_ = 0.18 ± 0.10). Of the 30 species that most declined in relative abundance (r_p-t_ < −0.36; Electronic Supplement ES2), all were classified as habitat specialists, occurring mainly in open, extensively used xerothermic grasslands. *Parnassius apollo*, *Hipparchia fagi*, *Colias palaeno*, *Iphiclides podalirius* and *Euphydryas maturna* (all r_p-t_ < −0.55) suffered the most severely (ES2).

**Table 2:** Major effects ANOVA of the difference Δp = p_2000_ − p_1800_ of relative abundances, and of the correlation r_p-t_ between the relative abundances *p* in study year *t* and the study year as dependent and species traits as predictor variables pointed to the degree of ecological specialization (specialists, generalists) to influence changes in relative species abundances.

Factor	df	Δp		r_p-t_	
		partial η^2^	P(F)	partial η^2^	P(F)
Family	6	0.12	<0.01	0.05	0.32
Biogeography	4	0.03	0.35	0.02	0.66
Diversity of habitats used	4	0.02	0.55	0.01	0.88
Habitat types	3	0.08	0.05	0.09	0.03
Diet breadth of the caterpillars	2	0.01	0.37	0.01	0.45
Dispersal	1	0.01	0.59	0.02	0.10
Degree of specialisation	1	0.07	<0.01	0.23	<0.0001
r^2^ (model)		0.38	<0.0001	0.49	<0.0001

Given are degrees of freedom (df_error_ = 123), partial η^2^ values, parametric significances P(F), and the coefficient of determination r^2^ of the whole model. As factors we consider the taxonomy (family), biogeography (western Palaearctic, continental, Mediterranean, alpine), diversity of habitats used, number of habitat types used, diet breadth of the caterpillars, dispersal behaviour, and degree of specialisation (generalist vs specialist).

Relative abundance in the 18^th^ century was not generally correlated with the future trend in abundance (r^2^ < 0.01; Fig. 2a). However, the relative abundance of the specialists in most cases decreased significantly while the one of the generalists increased. Even more, the higher the relative abundance of specialists in the 18^th^ century, the higher was the later likeliness of decrease of relative abundance, while the increase rate in relative abundance of generalists was without marked differences with respect to the initial abundance. The backward perspective confirmed these trends (Fig. 2b). Thus, generalist species in general had increased prior to the 21^st^ century, with abundant generalists (relative abundance p > 0.01) benefiting particularly, while specialist species had declined, with rare specialists (p < 0.001) being particularly affected (Fig. 2b). Additionally, all specialist species had relative abundances greater 0.1% before 1800 (Fig. 2a), while 48 of the 90 detected specialist species had a relative abundance of less than 0.1% in the 21^st^ century. In total, 103 species declined in relative abundance, while only 52 species increased (Electronic Supplement ES2).

Mobile species are mainly habitat generalists (one-way ANOVA: P < 0.001; Fig. 2c). Consequently, the changes in relative abundance were weakly linked to dispersal behaviour (Fig. 2c, Table 2). In general, mobile species tended to increase in relative abundance, whereas sedentary species were not (r^2^ = 0.06, parametric P < 0.01; Fig. 2c). There was a weak tendency of accelerating decrease rates in relative abundance of habitat specialists with increasing dispersal behaviour (r^2^ = 0.06, parametric P = 0.02; Fig. 2c). The relative abundances of species depending on xerothermic habitats (i.e. hot and dry habitats, such as stony slopes with sparse vegetation) and hygrophilic species (i.e. taxa depending on bogs and other wetlands) decreased, while the one of mesophilic species (i.e. taxa occurring in non-extreme habitats, where also the majority of nitrogen-tolerant or nitrogen-loving plant species are found) remained stable (Fig. 2d, Table 2). The few truly ubiquistic species (i.e. taxa using a large variety of different habitat types and resources) strongly increased in their relative abundances (Fig. 2d).

### Shifts in larval habitat requirements

Average habitat requirements of the butterfly and burnet moth species’ larval host plants confirmed the breakpoint in 1955. After 1955, the proportion of species associated with plants of higher light requirements, continentality (i.e. species depending on more continental climatic conditions, such as cold and dry winters, hot summers), and soil humidity increased significantly (P < 0.01) (Table 3, Fig. ES1A). Before 1955, we found an opposite trend for continentality (Table 3, Fig. ES1A), while the values for other habitat requirements remained constant.

**Table 3:** General linear model with all 115 time windows, 53 windows from 1750–1955, and 62 windows ≥1956 as dependent and average Ellenberg indicator values of larval host plants and of average butterfly dispersal ability as predictor variables.

Factor	All time windows			Windows <1956			Windows ≥1956		
	β-value	partial η^2^	P(F)	β-value	partial η^2^	P(F)	β-value	partial η^2^	P(F)
Light	0.00	<0.01	0.98	0.02	<0.01	0.92	0.33	0.12	<0.01
Temperature	0.25	0.06	<0.01	0.12	0.02	0.39	0.17	0.04	0.12
Continentality	0.05	<0.01	0.61	−0.43	0.17	<0.001	0.53	0.31	<0.001
Humidity	0.43	0.11	<0.001	−0.19	0.02	0.34	0.36	0.16	<0.001
pH	0.38	0.12	<0.001	−0.19	0.03	0.27	0.15	0.03	0.23
Nitrogen	0.12	0.01	0.29	0.13	0.01	0.42	0.04	<0.01	0.72
Dispersal	−0.13	0.02	0.18	−0.49	0.20	<0.001	0.47	0.26	<0.001
r^2^ (model)		0.26	<0.001		0.32	<0.001		0.58	<0.001

Given β-values for each predictor, partial η^2^ values, parametric significances P(F), and the coefficient of determination r^2^ of the whole model.

The division into communities up to and after 1955 is also traceable in the average Ellenberg scores of the main larval food plants of these two periods (Table ES1). Mean values for light (one-way ANOVA parametric P < 0.001), humidity (P = 0.001), pH (P < 0.001), and nitrogen (P < 0.001) significantly differed between both periods. While the average Ellenberg scores for light requirements of these plants were higher up to 1955 than afterwards, the scores for humidity and nitrogen were higher in the period since then. In a linear modelling, we did not find differences (at the 1% error level) between Ellenberg categories and the temporal trends of species abundances (Table ES1). Particularly, we did not find an increase in species associated with thermophilic plants (Ellenberg indicator values for temperature and humidity) and those of higher soil nitrogen concentrations (ANOVA parametric significances > 0.10; ES1A). We observed a weak tendency (r^2^ = 0.07; ES1A) for host plants of increased continentality.

## Discussion

In the time of dramatic loss of biodiversity worldwide, we did not find a significant decrease in species numbers over time when considering our entire study region in south-western Germany, neither for generalist nor specialist species. However, our results go in line with other large-scale assessments showing no significant or only marginal losses of species numbers^5,8,18,19^. In contrast, studies referring to more restricted areas or single sites revealed significant losses of species over time^1,3,6^, hence pointing at the high importance of the size of the study area if addressing the complete loss of species in an entire area^20,21^. As our study region covers a large geographic area, it consequently is not really surprising that almost all of the species are still observable, at least at some few localities. This is even less surprising as some high-quality nature reserves exist, being under permanent management, sometimes even explicitly for the conservation of specific butterfly and burnet moth species. Thus, these protected areas have apparently preserved some of the highly threatened species from final extinction, especially as land-use in them is likely in accordance with the ecological requirements of these target species.

However, although the rate of complete extinction in the entire study region so far is low, our data already show that the relative abundances of the majority of species have decreased significantly over time. This goes in line with the fact that most nature reserves across our study region are small, with a mean size of 84 ha, just covering 2.4% of the total surface^22^. Thus, populations living therein are assumed to be susceptible to population fluctuations and subsequent stochastic extinctions^23^. This situation (a large study region, but only small-sized and isolated habitat remnants, i.e. survival places for populations) may lead to a continuing decline in relative abundance of the majority of species, especially if being specialists, such as taxa depending on semi-natural grasslands or bogs. As a final consequence, even if mostly not having happened so far, a complete vanishing of species from our study region might happen in the future, maybe not that far from now. As an additional consequence of these shifts in relative abundances, todays´ communities are dominated by some few generalist species; as being generalists, these are using a large variety of habitat types and resources, and thus are able to respond more flexible on environmental changes as agricultural intensification and the vanishing of high quality habitats^24^.

The decrease in relative abundance of the majority of species observed in our study and the subsequent domination of the communities by a relatively small number of remaining habitat generalists is congruent with other studies on butterflies showing changes in the trait space of butterfly communities from specialist to generalist characteristics with increasing land-use intensity^25^. The herewith linked decrease in species evenness was also observed in another study on community shifts of temperate butterflies and burnet moths in a nature reserve in south-eastern Germany^3^. This development is fuelled by the fact that habitat specialists rely more tightly on specific habitat structures and resources during their pre-imaginal and imaginal stages than generalists^26^ and thus should suffer stronger under the consequences resulting from changing land-use, i.e. the deterioration of habitat quality^24^ and changes in landscape configuration, such as increasing habitat isolation^13^.

Furthermore, our data indicate family-specific responses to environmental changes (Table 3). Thus, many representatives of lycaenid and papilionid butterflies as well as zygaenid moths are particularly negatively affected showing the strongest reductions in their relative abundances. This might arise from the fact that the large majority of species belonging to these families require specific habitat structures (mainly extensively used grasslands), are in need of specific larval food plants and frequently show limited dispersal behaviour^23,26^. Thus, apart from the destruction of suitable habitats, the fragmentation of formerly interconnected habitats represents another important component for negative population trends^27,28^, as also mirrored in our relative abundance values. Consequently, as dispersal behaviour may be strongly impacted by landscape structures and resource availability, the realised movements of butterflies may strongly change depending on the respective environmental structures^29–31^. In contrast to most lycaenid butterflies and zygaenid moths, our data indicate increases in relative abundance for many species belonging to the family Pieridae with most of these species using a broad variety of resources (e.g. number of habitat types used and with respect to larval food plants)^26^ and consequently may be much better pre-adapted to environmental changes and the consequences of the still on-going monotonisation of our landscapes.

When addressing the temporal aspect of changes more in detail, our data show that relative abundances of species decreased particularly since the time after World War II, i.e. with the beginning of agricultural intensification. Consequently, these changes, most likely, only aggravated strongly over the past few decades (since the mid-1950ies until today); our data even let us argue that significant changes just started by that time. Comparable observations also exist for regions in western Europe, being well documented e.g. in the Netherlands^1^ and the UK^2^. Similar negative trends like for species diversity were recently recorded for losses of biomass of flying insects^8^. However, these decreasing species abundances, shifts in species community compositions and losses in biomass so far are mainly documented for agricultural landscapes, independently from being outside or inside of nature reserves^6,8^.

This coincidence between land-use intensification and accelerated changes of community compositions, with decreases in the relative abundances of specialist species, further support previous findings that indicate the negative effects from habitat destruction and habitat deterioration on biodiversity^24^. Although our data do not identify direct causes leading to these losses in relative abundances of the majority of butterfly and burnet moth species, a pronounced negative effect of the strong land-use intensification during the last decades on the large majority of butterflies and burnet moths and particularly on the specialist species is highly likely because both processes occurred roughly in the same time span.

Looking at our data more in detail, specialist species depending on xerothermic grassland ecosystems are the main losers^32^. This most likely is a response to large-scale habitat destruction of the formerly widespread and common habitat type of extensively used grassland areas (i.e. meadows and pastures) in our study region due to transformation into intensively used grasslands or even arable fields, or succession of former extensively used grasslands. Hence, our results for this specialised group of species are well reflecting the vanishing of species-rich meadows by about 80% since the 1940s^33^. This habitat loss is also mirrored by the changes in the ecological demands of the larval food plants (reflected by their Ellenberg indicator values) needed by the changing butterfly and burnet moth communities. Thus, the light demand of the respective larval food plants is lower since 1956 than before, reflecting the entire loss of such open and sunny habitats or their abandonment with subsequent encroachment of shrubs and bushes. Furthermore, the nitrogen demand of the needed food plants has increased thus going in parallel with the general eutrophication of the landscape and the loss of nutrient poor grasslands in particular. Finally, the humidity demand of the food plants is higher in the recent period if compared with the ancient one. This underlines that in particular the dry calcareous grasslands, representing a rather characteristic habitat type of our study region, are seriously affected by decline.

Seeing this entire problem more generalised, it needs to be emphasized that at least three fundamental steps in changes of land-use intensification since World War II have been identified by several studies^34–36^: (i) land consolidation mostly since the 1970s with subsequent landscape (and habitat) homogenization; (ii) increasing application of pesticides mostly since the 1980s, including highly efficient chemical agents such as neonicotinoids; and, in parallel, (iii) increasing atmospheric nitrogen deposition (mainly from traffic, industry and households, but also from agriculture). Various studies showed that the consequences resulting from these changes negatively impact biodiversity and should be seen as the main drivers of insect decline as recently reviewed by Habel and colleagues^24^. Additionally, most of the remaining suitable habitats are comparatively small and geographically isolated from each other, and thus habitat quality may further decrease due to negative edge effects, such as drifting pesticides and direct nitrogen influx from nearby arable fields^3^. Therefore, based on this knowledge on general environmental changes but also on our data presented in this article, we plea for an immediate change in EU agricultural policy to avoid further losses of butterflies, and biodiversity in general. In concrete, we advocate for: (i) an increase of landscape heterogeneity, including more flower-rich habitats^37^; (ii) an increase of organic farming^38,39^; (iii) a complete ban of aggressive pesticides; and, (iv) environmental taxing of products causing high nitrogen deposition (e.g. meat from mass production).

## Methods

### Data set

We compiled data on butterfly (Rhopalocera and Hesperiidae, with the families Hesperiidae, Lycaenidae, Nymphalidae, Papilionidae, Pieridae, Riodinidae) and burnet moth (Zygaenidae) species occurrences, which were collected from lepidopterists across an area of 35,750 km^2^ in south-western Germany (i.e. the federal state of Baden-Württemberg). A map showing the location of this study area is provided in Electronic Supplement ES3). The period of data collection covers a time span of 268 years. This data set consist of data from records from historical field books, protocols, yearly reports, index cards, literature, diaries and butterfly collections, as well as various records memorized in MS Access electronic data bases (InsectIS, www.insectis.de, www.schmetterlinge-bw.de). These data were previously checked and adjusted by various lepidopterists working in this region since decades, and subsequently compiled for various books and scientific articles^40–43^. The data set used in this study consists of 233,307 single records and represent 155 butterfly and burnet moth species. An overview of data sets and collectors is provided in Electronic Supplement ES4. We excluded information on local species abundance due to the fact that data collection has been performed without any standardized methodology. A complete list of all data is given in Electronic Supplement ES5.

### Functional traits

For each species, we assigned information on its habitat requirements and behaviour. We considered the following variables: Number of habitat type(s) used (considering: ubiquist, mesophilic-generalist, mesophilic-open land, mesophilic-with shrubs, mesophilic-forest, xerothermophilic-in general, xerothermopilic-open land, xerothermophilic-with shrubs, hygrophilic, tyrphostene, alpine); diversity of habitats used (considering: one single main habitat type, several habitats used within the same habitat complex, all habitats within the same complex, various habitat complexes); diet breadth of the caterpillars (considering monophagous: host plants from one plant genus, oligophagous: host plants from one plant family, polyphagous: host plants from several plant families); dispersal behaviour (considering: extremely sedentary, sedentary, mostly sedentary, little sedentary, dispersive, mostly migratory, migratory, extremely migratory); generalist/specialist (general classification based on all parameters described above). For this latter general classification, we grouped parameters into three levels of specialization (1–3, with increasing level of specialization) and calculated mean values. Species with means <1.5 were interpreted as generalists, and species >1.5 as specialists. Data for species classification were taken from the literature^26,44,45^. In addition, we considered main larval host plant(s) used by the larvae of the lepidopterans in our study region. We assigned Ellenberg indicator values^46^ (light, temperature, continentality, humidity, pH, nitrogen) to each of these food plants. A complete list of all functional traits and the respective Ellenberg indicator values of the main larval food plants are given in Electronic Supplement ES2.

### Statistical analysis

#### Data base

Due to the fact that sampling intensity and spatial and temporal coverage varied strongly over time and the level of quantitative sampling increased significantly across time (Table 1), a direct comparison of annual samples (in terms of abundances) was not possible (see also^47,48^). Therefore, we here only compare proportions of species with respect to ecological characteristics.

We first organized the data in a 155 species × 173 study year matrix containing the number of records of each species for each year (Electronic Supplement ES5). We calculated the relative abundance of each species in a given study year as the number of records of the focal species divided by the total number of records in this year. Differences in the number of records depend on several factors, particularly differences in sample sizes, sample biases towards rare species, natural annual variability in community composition, and changes in environmental conditions, to name the most important. In the present case, sample size (the total number of species records) strongly increased with time (Table 1, Fig. ES1Ca), possibly masking underlying changes in species richness and composition. Species richness increased with sample size (Fig. 2b) in line with the species–sample size relationship *SSR*^49^. Too low sample sizes cause a fast decrease in richness indicating severe undersampling. Therefore, we applied a breakpoint analysis^50^ and moved an assumed breakpoint along the time series of a response variable and determined the steepest changes in the slopes of two linear regressions between response variable and time below and above the assumed breakpoint (Fig. ES1Cb). This analysis identified an initial exponential increase in richness with sample size (ln-transformed number of records). Above 115 annual records, this increase changed to a logarithmic increase (Fig. ES1Cc) being in line with observed empirical SSR patterns. To obtain this breakpoint of 115 records, we pooled years into windows of 50 years (1751–1800, 1801–1850), 20 years (1851–1870) and 10 years (1871–1880, 1881–1890, 1891–1900, 1901–1910) and added up the respective samples. In all other study years, sample sizes were above the breakpoint. The resulting final 155 species × 115 time window matrix containing the number of records per species and time window is contained in the Electronic Supplement ES5.

#### Temporal trends in species richness, composition, and abundance

To assess temporal changes in species richness, relative abundances, and composition, we applied two approaches. We first used multiple random samples to assess expected species richness for any given time window. Precisely, we assigned to each time window *i* (having *n*_*i*_ records) 100 random samples of size *n*_*i*_ each from all other time windows where more specimen had been recorded. With this technique, we accounted for the variation in the relative abundances among the time windows to obtain long term averages in expected richness. Our method appears to be superior to rarefaction and single random sample approaches that are heavily dependent on the distribution of species abundances that defines the (arbitrary) baseline for sampling.

This sampling procedure resulted in a non-linear relationship between predicted richness and sample size (Fig. ES1Cc) according to a Gompertz statistical distribution, which is frequently applied in studies with high failure rates^48^. From these samples, we obtained for each window the average expected species richness (S_i,exp_) and the respective standard deviations (σ_exp_) and calculated the effect sizes (ES = S_i_ − S_i,exp_) and the standardized effect sizes (SES = ES/σ_exp_) as indicators of whether the species richness in a given study year was higher or lower than expected from multiple random samples (Fig. ES1D).

In the second approach, we compared the relative abundances (p = n_i_/N, where N is the total number of records per time window) of each species. We used Pearson correlation r_p-t_ of relative abundance and time to assess tendencies of increase and decrease in relative abundance. We used general linear modelling to relate these correlations to the average observed relative abundance in the first (1750–1800) and last time windows (2000–2017), to larval host plant habitat conditions (average Ellenberg values for light, temperature, continentality, humidity, pH, and nitrogen), and to dispersal ability. We calculated these average scores from the inner products of the trait × species and the species × time window matrices divided by the number of species per window. In addition, we also analysed potential temporal trends without considering any time steps. Finally, we used analyses of variance to relate differences in species abundances and in the temporal trends in abundance (r_p-t_) to species traits and family membership.

## Supplementary information

Electronic supplement ES1: Long-term large-scale decline in relative abundances of butterfly and burnet moth species across south-western Germany

Electronic supplement ES2: Overview of all species traits used for analyses with respective classifications.

Electronic supplement ES3: Location of our study area (small inlet map, marked in grey), and the grid cells covering our study area.

Electronic supplement ES4: Overview of all data sets used in this study, with details about sources and observers.

Electronic supplement ES5: Overview of all species, with the number of observations of single years.
