# Supplementary material for: Long-term large-scale decline in relative abundances of butterfly and burnet moth species across south-western Germany
Source: Sci Rep. 2019 Oct 17;9:14921. doi: 10.1038/s41598-019-51424-1 (PMC6797710; doi:10.1038/s41598-019-51424-1)
Supplement: Supplementary file 1 — Electronic supplement ES1: Long-term large-scale decline in relative abundances of butterfly and burnet moth species across south-western Germany [file 41598_2019_51424_MOESM1_ESM.docx]

**Electronic supplement ES1**

**Long-term large-scale decline in relative abundances of butterfly and burnet moth species across south-western Germany**

Jan Christian Habel, Robert Trusch, Thomas Schmitt, Michael Ochse, Werner Ulrich

Table ES1: Major effects ANOVA did not find significant relationships between the habitat requirements of butterfly larval host plants (quantified for each species by the associated Ellenberg scores) and the correlations between study window and relative abundance r_p-t_, the difference in relative abundance **Δ***p* = *p*(>2000) – *p*(1750), and the species relative abundances *p* 18^th^ and the 21^th^ centuries.

|  |  | **r_p-t_** | | **Δp** | | **p(1750)** | | **p(>2000)** | |
| --- | --- | --- | --- | --- | --- | --- | --- | --- | --- |
| **Factor** | **df** | **partial** η**^2^** | **P(F)** | **partial** η**^2^** | **P(F)** | **partial** η**^2^** | **P(F)** | **0.01** | **P(F)** |
| **Light** | 7 | 0.01 | 0.42 | <0.01 | 0.55 | 0.01 | 0.47 | <0.01 | 0.78 |
| **Temperature** | 4 | <0.01 | 0.60 | <0.01 | 0.57 | <0.01 | 0.65 | 0.01 | 0.41 |
| **Continentality** | 5 | 0.07 | 0.04 | 0.06 | 0.07 | 0.05 | 0.16 | 0.10 | 0.02 |
| **Humidity** | 9 | <0.01 | 0.91 | <0.01 | 0.97 | 0.08 | 0.08 | <0.01 | 0.49 |
| **pH** | 8 | <0.01 | 0.50 | <0.01 | 0.56 | <0.01 | 0.56 | <0.01 | 0.58 |
| **Nitrogen** | 8 | 0.01 | 0.48 | 0.04 | 0.10 | <0.01 | 0.58 | 0.03 | 0.17 |
| **r^2^ (model)** |  | 0.10 | 0.38 | 0.11 | 0.33 | 0.17 | 0.32 | 0.15 | 0.15 |

Figure ES1A. Temporal changes of average Ellenberg values of Butterfly larval host plants for 53 time windows from 1750–1955 (red data points and linear regression line) and 62 windows ≥1956 (blue data points and black regression line). Coefficients of determination r^2^ > 0.13 are parametrically significant at P < 0.01.

Figure ES1B. Temporal changes of average dispersal ability of butterfly species for 53 time windows from 1750–1955 (red data points and linear regression line) and 62 windows ≥1956 (blue data points and black linear regression line). Coefficients of determination r^2^ > 0.13 are parametrically significant at P < 0.01.

Figure ES1C: Sample intensity significantly increased in time (a). Observed (b) and predicted (c) species richness in dependence of ln-transformed total numbers of records. Richness predictions in (c) are averaged random samplings from all study years with higher number of records than in the focal year. Full and open data points in (b) were separated from breakpoint analysis maximizing the explained variances of both regressions (full data: exponential model, open data: linear model). The breakpoint separates study years above and below 113 records. Explained variances (r^2^) refer to ordinary least squares. Error bars in (c) denote one standard deviation. The red regression line in (c) denotes the fits of the Gompertz model:

$S_{i,exp}=155.44e^{-9.292e^{-0.46ln(n_{i})}}$ (1)

Where S_i,exp_ is the expected number of species and n_i_ is the total number of records (sample size) in year i.

Figure ES1D. Effects sizes of the random sample model for low (red regressions and explained parametric variance) and high (green) total numbers of records (a), observed total species richness (b), and study years (c). The green regressions (a, c: linear, b: quadratic) are parametrically significant at P < 0.001. The linear red regressions are insignificant at P > 0.10.
