# Supplementary material for: Long-term large-scale decline in relative abundances of butterfly and burnet moth species across south-western Germany
Source: Sci Rep. 2019 Oct 17;9:14921. doi: 10.1038/s41598-019-51424-1 (PMC6797710; doi:10.1038/s41598-019-51424-1)

**Electronic supplement ES3**

**Long-term large-scale decline in relative abundances of butterfly and burnet moth species across south-western Germany**

Jan Christian Habel, Robert Trusch, Thomas Schmitt, Michael Ochse, Werner Ulrich

Location of our study area (small inlet map, marked in grey), and the grid cells covering our study area.


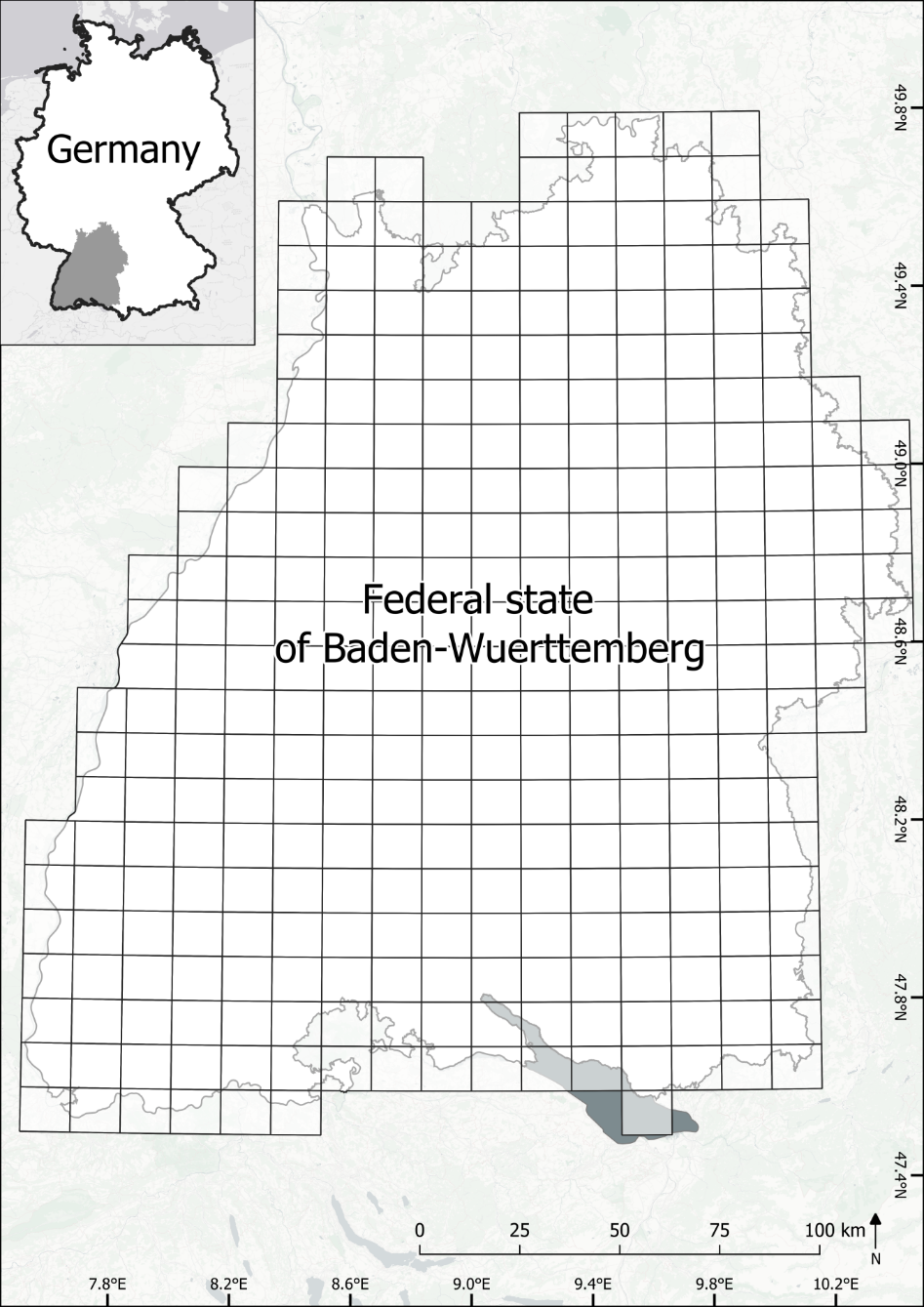

Supplement: Supplementary file 3 — Electronic supplement ES3: Location of our study area (small inlet map, marked in grey), and the grid cells covering our study area. [file 41598_2019_51424_MOESM3_ESM.docx]
